# An Uncommon Presentation of an Uncommon Bone Tumor: A Case Study of a Pathologic Fracture of an Intertrochanteric Aneurysmal Bone Cyst

**DOI:** 10.7759/cureus.6461

**Published:** 2019-12-25

**Authors:** Matthew G Weber, Juston Fan, Ryne Jenkins

**Affiliations:** 1 Orthopaedic Surgery, Riverside University Health System Medical Center, Moreno Valley, USA

**Keywords:** abc, pathologic fracture, intertrochanteric femur lesion, orthopedic surgery

## Abstract

Aneurysmal bone cyst (ABC) is a benign, destructive lesion characterized by a expansile fluid-filled cystic structure primarily affecting children and young adults. Common treatment modalities include arterial embolization, curette, intralesional injections and en bloc resection with instrumentation placement. We present the case of a 22-year-old patient presenting to the emergency department with an ABC in the intertrochanteric region of the right femur and a minimally displaced pathologic femoral neck fracture. Open biopsy with curettage, bone grafting and cephalomedullary nailing were performed with fracture stabilization and favorable recovery. Reports of these lesions presenting with pathologic fracture are scarce. We discuss treatment modalities and guidelines for ABCs and pathological fractures. Future studies are needed to assess clinical guidelines for the management of ABCs and pathological fractures.

## Introduction

Aneurysmal bone cysts (ABCs) are benign, osteolytic, destructive lesions with potential to metastasize, which is characterized by expansile fluid-filled cystic structures that can cause pain. Most studies suggest the incidence of ABCs is 0.14 per 100,000 people. This accounts for 1% to 6% of all primary bone tumors with 80% presenting within the first two decades of life and a median age of 13-15 years old [1]. In fact, 75% of patients are younger than 20 years. ABCs primarily occur in the metaphysis of long bones with highest incidence occurring in the femur [2]. Most of these lesions are considered primary lesions, arising de novo, but up to 30% are found to be associated with other benign and malignant tumors, most commonly giant cell tumors of bone [3]. The etiology and nature of this lesion is poorly understood. Some theories suggest that lesions arise from vascular malformations, trauma or genetics [4]. Primary ABCs have been shown to be true neoplasms linked with chromosomal fusion that may affect osteoblastic maturation. In fact, one study showed that the TRE17/USP6 locus occurs in over 60% of ABC cases resulting in TRE17 overexpression ultimately blocking osteoblastic maturation [5]. Secondary ABCs can also arise from other neoplastic lesions including giant cell tumors, osteoblastomas, osteosarcomas and chondroblastomas [3].

Common treatment modalities include arterial embolization, curettage, intralesional injections, cryotherapy, sclerotherapy and en bloc resection with instrumentation placement [6,7]. Curettage and bone grafting are the current standards of practice along with or without additive treatments [3]. ABCs very rarely present with pathologic fracture. Treatment options for pathological fractures primary include placement of an intramedullary nail, plates or prothesis with respective advantages and disadvantages based upon lesion region [8]. Overall recurrence rate following surgical treatment alone is about 20% [9]. With the addition of surgical techniques such as cryotherapy or sclerotherapy, recurrence rates have been shown to be as low as 3%.

## Case presentation

**Figure 1:**
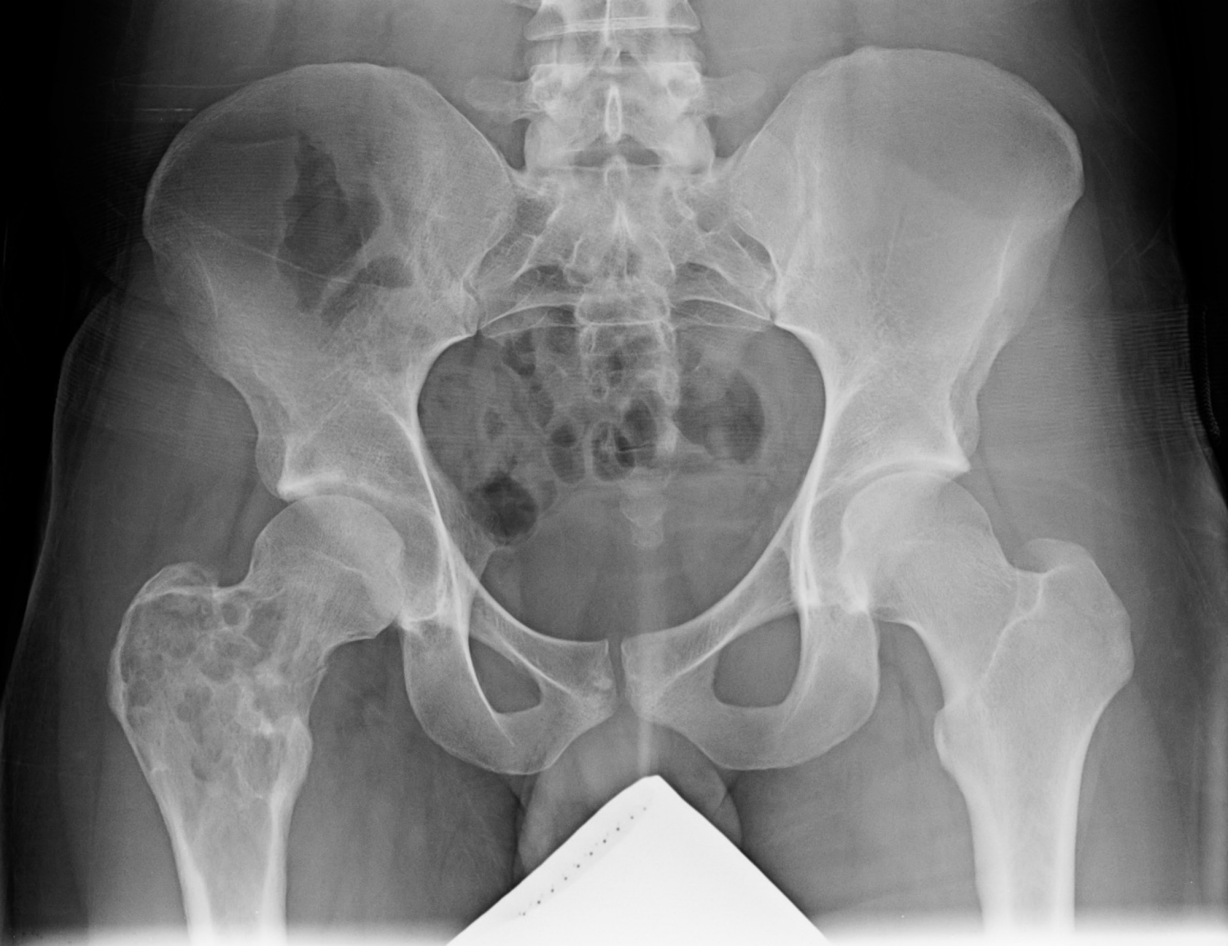
Anterior Posterior Pelvis Radiograph Anterior posterior pelvis radiograph demonstrating a lytic bone lesion within the intertrochanteric region of the right femur. Note the characteristic "soap-bubble" appearance. There is also a subtle non-displaced pathologic femoral neck fracture.

**Figure 2:**
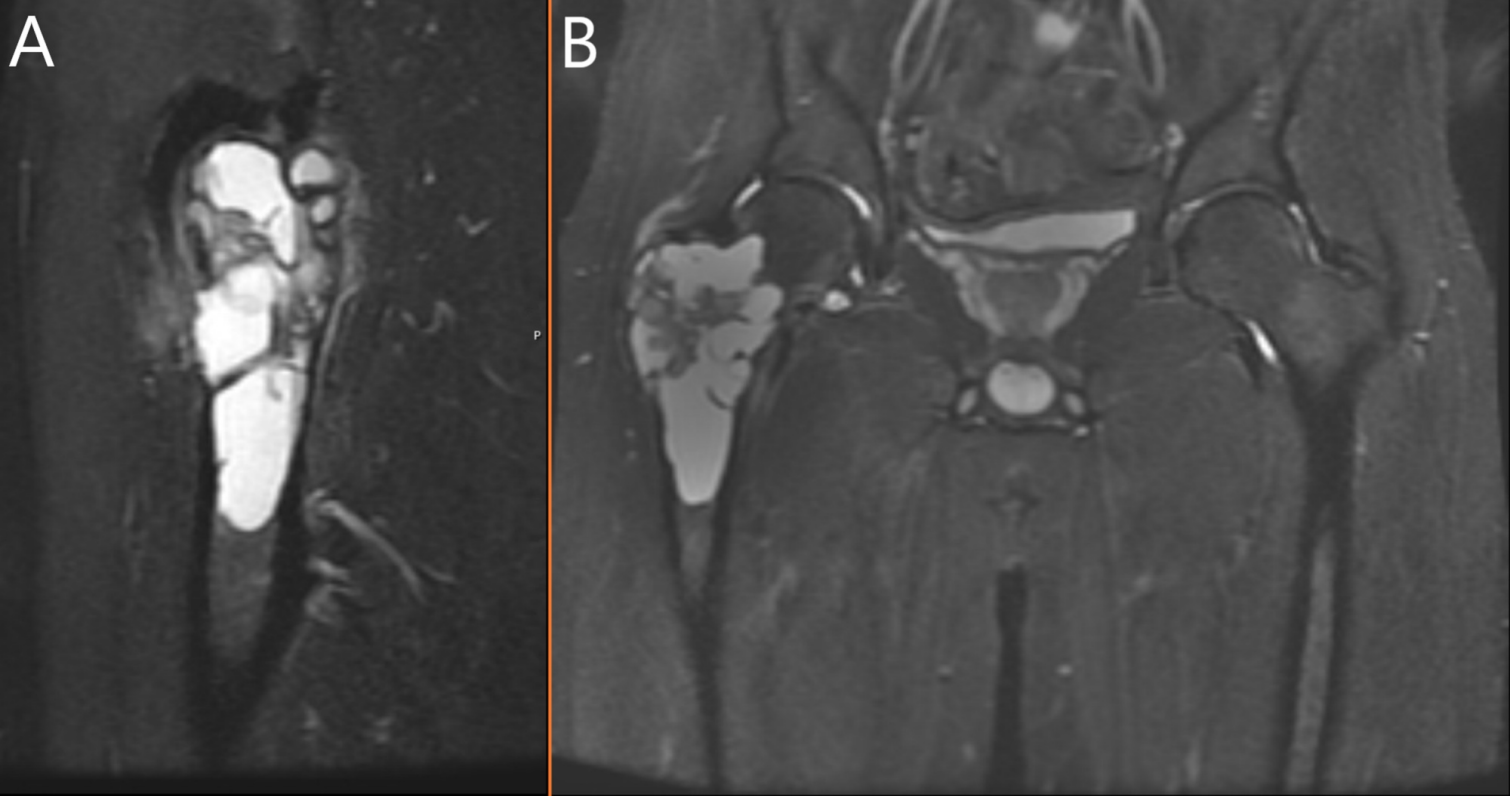
MRI STIR Sequence Images Sagital (A) and coronal (B) MRI STIR sequence images of the lesion. Note the fluid-fluid levels present in these planes. The "bubbly" appearance of this tumor arises from bony septa which divide the blood-filled caverns.

An otherwise healthy 22-year-old male presented to the emergency department (ED) with two months of low back pain. The patient described the pain as worsening and localized to the right hip. He had been seen by various other healthcare facilities for chronic “low back pain” that waxed and waned. He had been taking ibuprofen and Tylenol without relief. He came to the ED after missing about two weeks of work secondary to the inability to bear weight on his right lower extremity following a near-fall incident. Admission radiographs demonstrated a lytic bone lesion within the intertrochlear region of the right femur. Characteristic "soap-bubble" lesions were visualized along with a subtle non-displaced pathologic femoral neck fracture (Figure 1). MRI images showed the classic “soap-bubble” appearance with “fluid-fluid levels” suggestive of an ABC (Figure 2). The patient denied any family history of musculoskeletal tumors.

**Figure 3:**
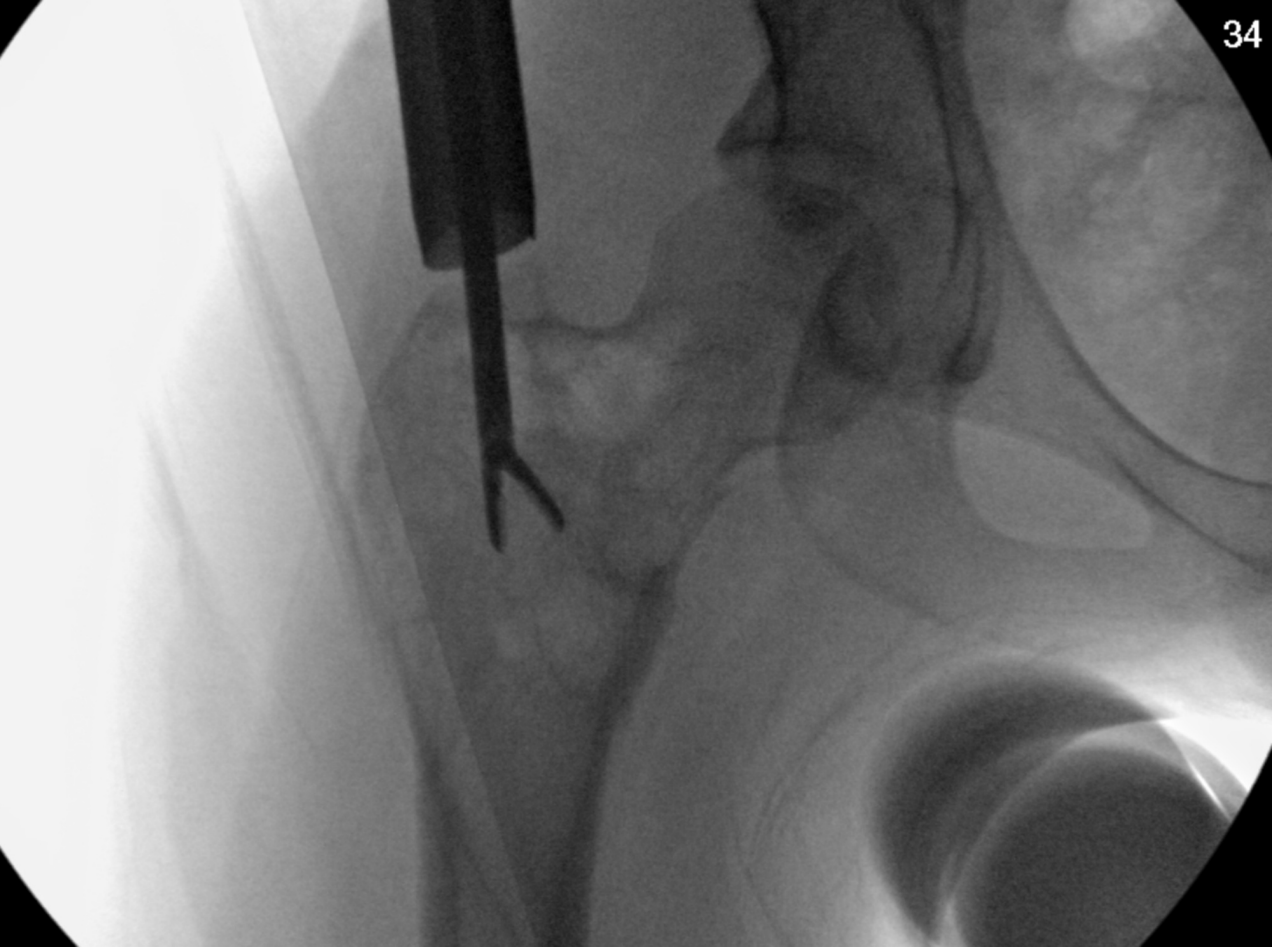
Intraoperative Fluoroscopic Confirmation of Biopsy Intraoperative fluoroscopy demonstrating intralesional biopsy.

**Figure 4:**
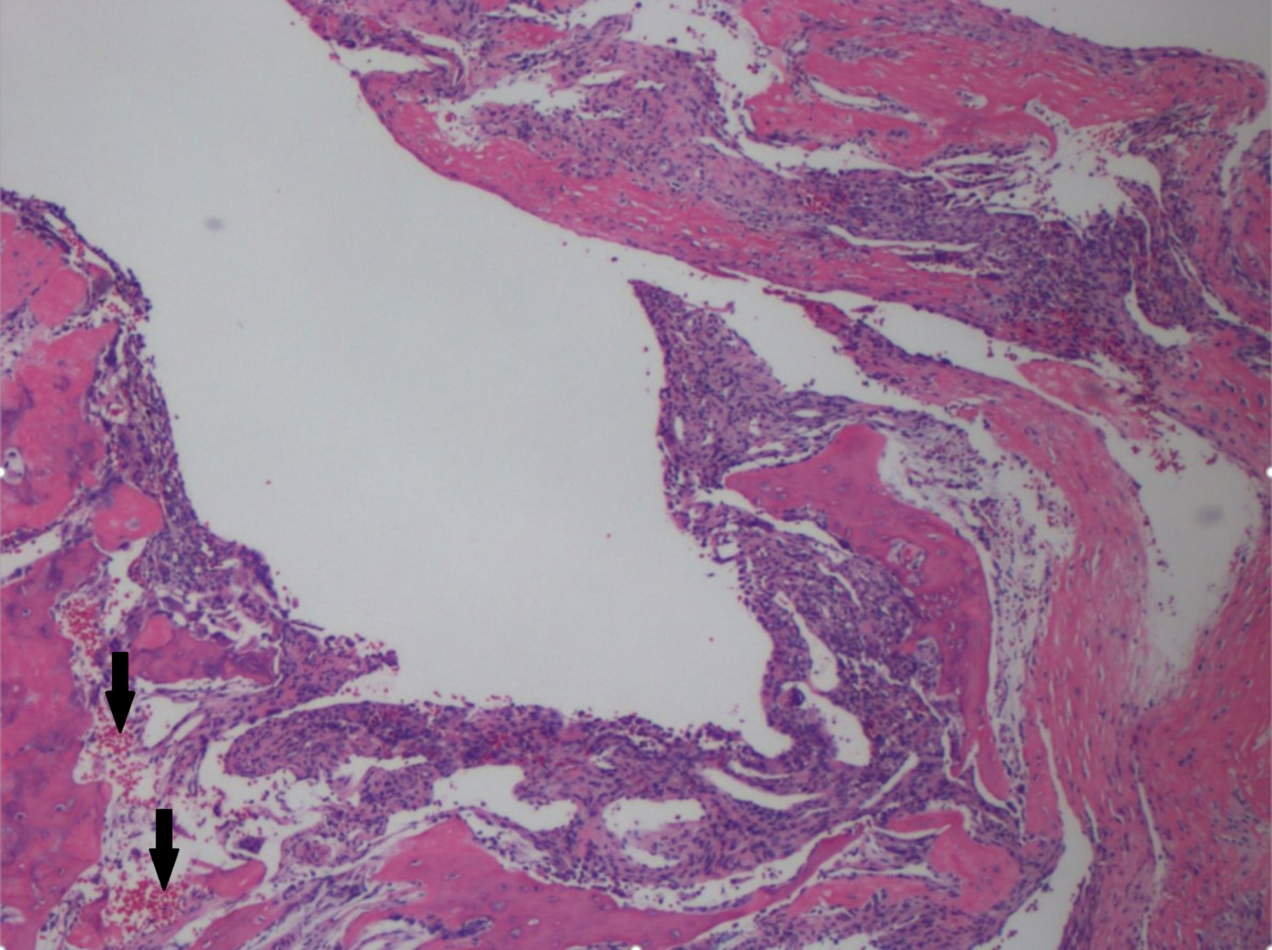
H&E Stain at 40x 40x, hematoxylin and eosin (H&E) stain, characteristic appearance of blood-filled cystic spaces without endothelial lining (black arrows).

**Figure 5:**
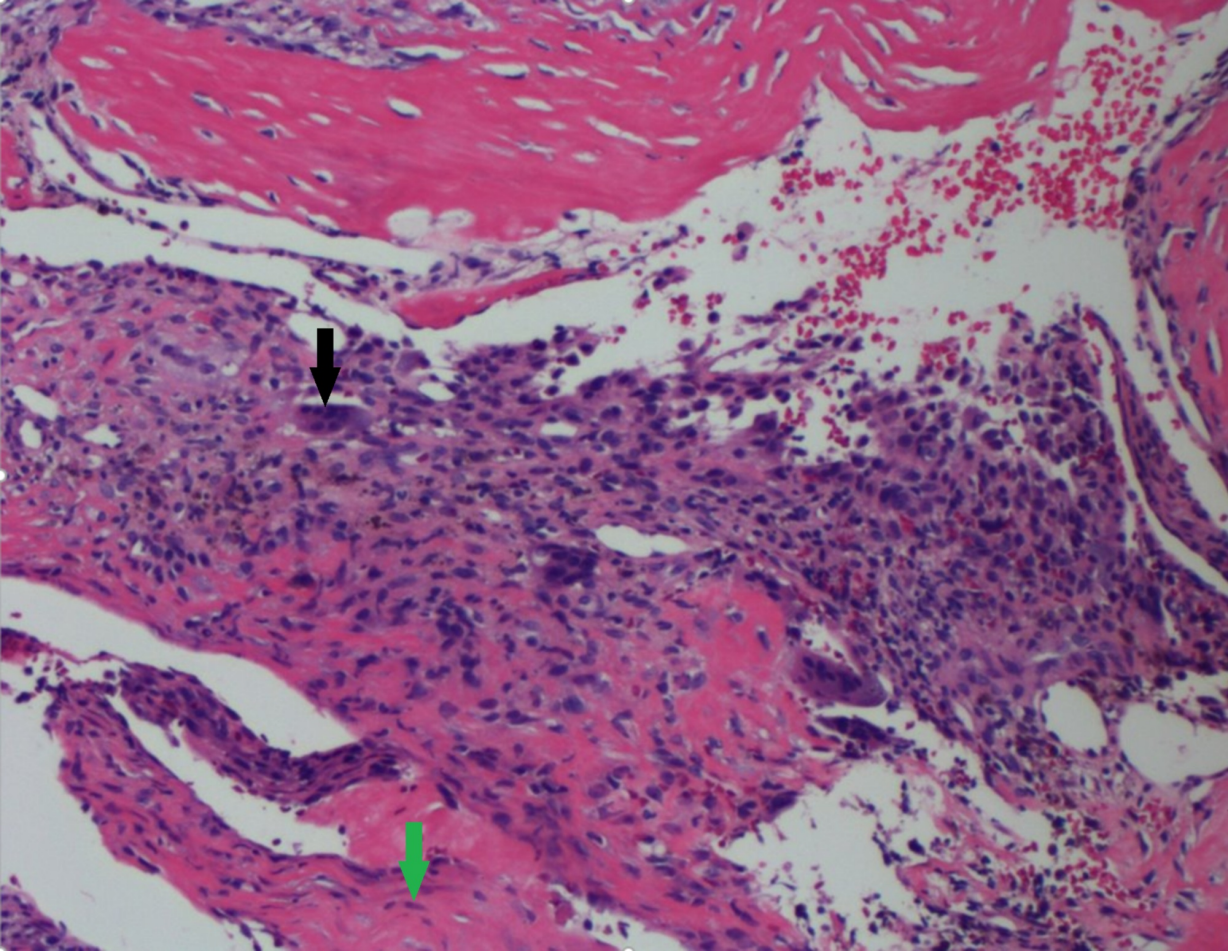
H&E Stain at 100x 100x, hematoxylin and eosin (H&E) stain, benign giant cells (black arrow), fibroblasts (green arrow), and thin strands of woven bone.

After a discussion with hospital radiology, it was concluded that this was certainly an ABC and would not require a biopsy prior to definitive fracture fixation for diagnosis confirmation. It was decided to proceed with an open biopsy, curettage with bone grafting and cephalomedullary nailing for his minimally displaced pathologic femoral neck fracture (Figure 3). Additional adjuvant therapy such as sclerotherapy was decided against due to the patient’s age and likely decreased risk for recurrence. Open biopsy demonstrated characteristic appearance of cyst without endothelial lining as well as benign giant cells, fibroblasts and thin strands of woven bone (Figures 4, 5).

**Figure 6:**
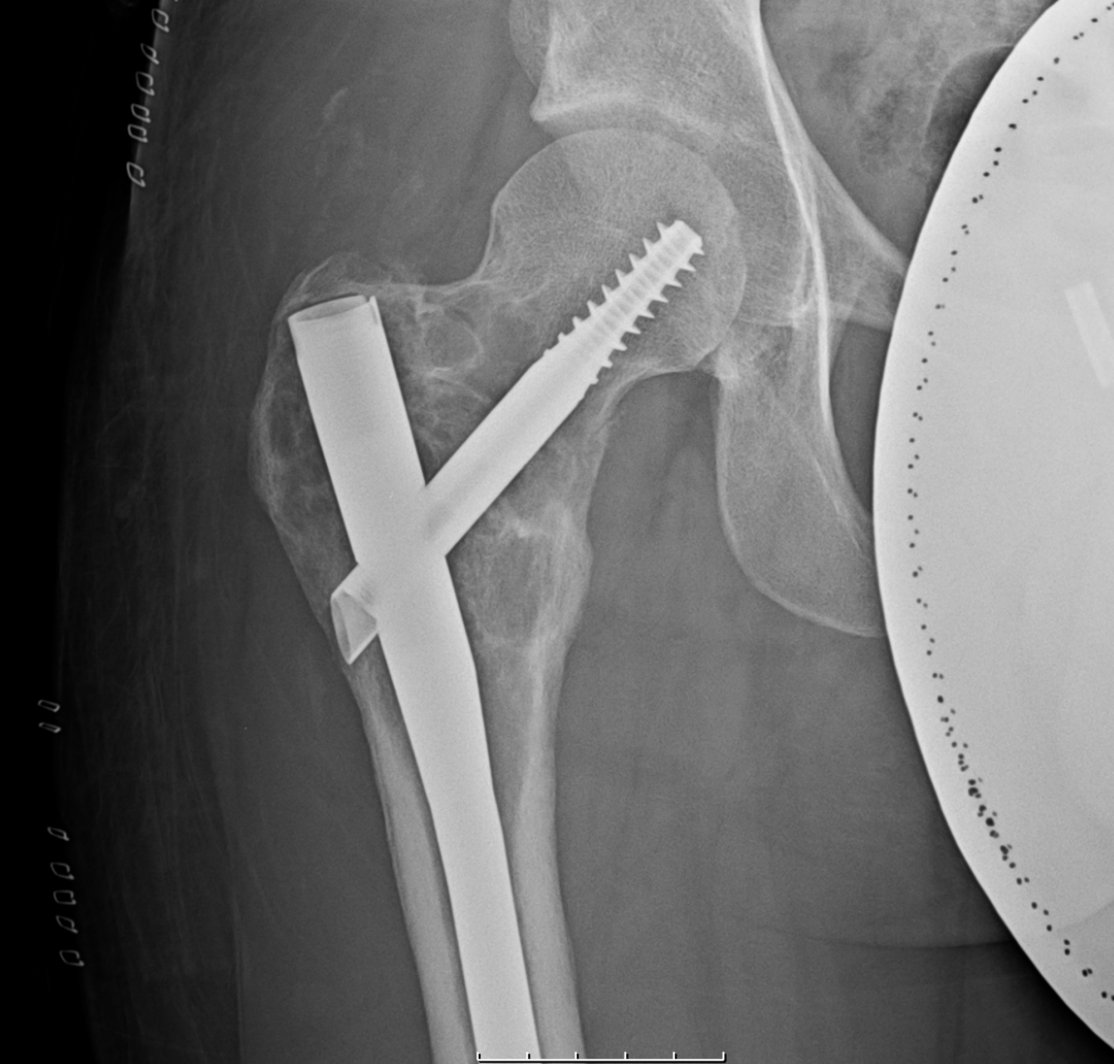
Post-operative Radiographs Radiographs taken two weeks post-operatively demonstrating the long-locked cephalomedullary nail stabilization of the fracture without interval displacement.

Post-operatively, the patient ambulated immediately with crutches for two weeks, and without assistance thereafter. X-rays taken two weeks post-operatively demonstrated stabilized fracture without interval displacement (Figure 6). He returned to work at three weeks post-operatively with significantly improved symptoms. Follow-up at six and nine months shows the patient was asymptomatic without recurrence of lesion.

## Discussion

We presented a rare case of a young adult presenting with an intertrochanteric ABC with a pathologic fracture of the femoral neck. Reports of these lesions presenting with pathologic fracture are scarce. When diagnosed with associated fracture, many of these lesions are treated non-operatively until the fracture heals. The standard treatment is curettage and bone grafting [3]. In this particular instance, internal fixation was indicated due to the existing fracture through a crucial weight-bearing region. Although other bony tumors can be associated with this lesion, pre-operative biopsy was deemed unnecessary, as review of the MRI by radiologists was sufficient to rule out the possibility of a malignancy. Therefore, our patient underwent open biopsy at the time of curettage, bone grafting and fracture fixation. There is a paucity of literature exploring the incidence of pathologic fractures associated with ABCs and even less of best standard treatment options. The rarity of this bone tumor and uncommon presentation makes the diagnosis difficult.

Treatment guidelines are debatable; however, currently, curettage along with bone graft or polymethylmethacrylate to induce ossification of bone lesion is the principal modality used [3,10]. Overall recurrence rate following surgical treatment alone is about 20% [9]. The use of cryotherapy in addition to curettage has been showed to reduce recurrence rates to only 3% to 5% [10,11]. Sclerotherapy has also been shown to significantly reduce recurrence rates to as low as 3% [12]. Adjuvant therapy such as argon beam coagulation has been shown to decrease recurrence as well [9]. Additionally, curettage with supplemental autologous bone marrow injections has been shown to augment bone healing [13]. A biopsy technique known as a "curopsy" or a percutaneous limited curettage at the time of biopsy has been shown to successfully resolve ABCs in 81% of patients [7]. This technique may be useful in decreasing the need for a second procedure following biopsy. Large bone defects following curettage require bone grafts to provide stability and structural support. Vascularized bone grafts have been proposed as the ideal graft for long-term viable reconstruction in pediatric patients; however, it is highly technical in nature and requires extensive resources [14]. Additionally, literature shows that non-vascularized, autologous bone graft can provide stable structural support for weight bearing with successful long-term results and, therefore, may be a better choice [15].

Treatment options for pathological fractures primarily include intramedullary nails, plates or prothesis with respective advantages and disadvantages based on region of bone lesion. In the case of our patient, surgical options for lesions involving the trochanteric region include intramedullary nails and prosthetic reconstruction. Studies specifying superiority are lacking, and the issue remains debated amongst physicians. Currently, the decision is based on the quality of bone stock. An intramedullary reconstruction nail including femoral neck and head fixation should be used in patients with sufficient bone stock. Patients with large lesions or insufficient bone stock should be treated with proximal femur modular tumor prosthesis [8]. ABCs primarily affect teenagers with median age between 13 and 15 years. This may serve as an issue for patients requiring fixation of fracture sites without complete closure of epiphyseal plates. Instrumentation with expanding shafts or revision surgeries may be needed to accommodate asymmetries in height and angles of alignment.

Non-surgical options may be indicated in areas where the lesion is inaccessible. Intralesional injections of calcitonin with steroids have proved to be safe and effective in regression of ABC lesions [16]. Doxycycline, Ethibloc® (Ethicon, Norderstedt, Germany), which is a mixture of zein, oleum papaveris and propylene glycol, and an aqueous solution of calcium sulfate have proved to be successful alternative treatments with low recurrence rates [17,18]. Radiotherapy is generally contraindicated secondary to adverse effects of radiation on growth plates and potential for future tumors. Additionally, oral bisphosphates and denosumab have proved to be effective treatments in symptomatic unresectable benign bone tumors [7,19]. Two studies assessing selective arterial embolization with N-2-butyl cyanoacrylate showed positive outcomes in 82% to 94% of patients after single and multiple embolization treatments [20]. This may prove as an effective and less invasive means of treatment as it is easily repeatable and can be done at a lower cost than surgery. With a high success rate and low rate of complications, this may serve as good starting treatment for patients who are hesitant or unable to undergo surgery.

## Conclusions

Curettage with bone grafting and cephalomedullary nail fixation provided a favorable outcome in our young adult patient suffering from a pathological fracture secondary to an ABC. Future studies of the clinical presentation, progression and incidence of pathologic fractures secondary to ABCs are warranted to better elucidate the nature of this disease process in order to optimize surgical treatment outcomes. Furthermore, multicenter clinical trials are needed to assess treatment superiority and general guidelines for management.
